# Bortezomib sensitizes human glioblastoma stem cells to adoptive natural killer cell cytotoxicity

**DOI:** 10.1186/2051-1426-3-S2-P17

**Published:** 2015-11-04

**Authors:** Steven Grossenbacher, Erik Ames, Stephanie Mac, Yuyou Duan, Syed Azeem, Robert Canter, William Murphy

**Affiliations:** 1 University of California, Davis, Sacramento, CA, USA

## Background

Glioblastoma multiforme (GBM) is the most common primary malignant human brain tumor and one of the most lethal of all cancer types. Despite the introduction of numerous non-surgical therapies for GBM, clinically relevant responses to treatment are rarely durable. Small populations of stem cells residing within GBM tumors have the ability to differentiate into diverse cell types *in vitro* and *in vivo*, and form new heterogeneous tumors in immune compromised mice. These cells have been found to express high amounts of aldehyde dehydrogenase (ALDH), can resist radiation therapy and chemotherapy, and represent an important target in the advancement of glioblastoma treatment.

## Methods

We and others have identified natural killer (NK) cells as having an inherent ability to kill human GBM stem cells (GSCs), through the recognition of specific activating proteins on the GSC surface which facilitate NK cell, activation, recognition, and killing. Here we found that primary human GBMs contain a small population of cells (4-14%) expressing high amounts of the stem cell associated protein ALDH which can be selectively killed by activated human NK cells.

## Results

Bortezomib, a small molecule proteasome inhibitor, has previously been found to sensitize human and mouse tumor cells to TRAIL and/or NK cell-mediated killing through the expression of apoptosis-inducing death receptors such as DR5. Additionally bortezomib has proved to have some anecdotal efficacy for adults with recurrent gliomas in early clinical trials. In this study we found that bortezomib significantly improves activated human NK cell killing of ALDH^bright^ human GSCs. Additionally we found that *in vitro* bortezomib treatment significantly enhances human GSC expression of Fas and DR5, while having a much smaller effect on ALDH^dim^ cells. Lastly, utilizing an orthotopic xenograft model of human GBM in the brains of NSG mice, we found that the administration of bortezomib prior to intracranial human activated NK cell infusions led to significant increases in tumor growth delay compared to either bortezomib or NK cells alone.

## Conclusions

These data provide preclinical rational for the combined use of bortezomib and adoptive NK cell therapy for the treatment of human GBM, with the potential to kill ALDH^bright^ GSCs.

